# Evaluation of non-inferiority of intradermal versus adjuvanted seasonal influenza vaccine using two serological techniques: a randomised comparative study

**DOI:** 10.1186/1471-2334-10-134

**Published:** 2010-05-26

**Authors:** Pierre Van Damme, Robert Arnou, Froukje Kafeja, Anne Fiquet, Patrick Richard, Stéphane Thomas, Gilles Meghlaoui, Sandrine Isabelle Samson, Emilio Ledesma

**Affiliations:** 1 University of Antwerp, Wilrijk, Belgium; 2 Reseau ALTI, Angers, France; 3 Sanofi Pasteur MSD S.N.C., Lyon, France

## Abstract

**Background:**

Although seasonal influenza vaccine is effective in the elderly, immune responses to vaccination are lower in the elderly than in younger adults. Strategies to optimise responses to vaccination in the elderly include using an adjuvanted vaccine or using an intradermal vaccination route. The immunogenicity of an intradermal seasonal influenza vaccine was compared with that of an adjuvanted vaccine in the elderly.

**Methods:**

Elderly volunteers (age ≥ 65 years) were randomised to receive a single dose of trivalent seasonal influenza vaccine: either a split-virion vaccine containing 15 μg haemagglutinin [HA]/strain/0.1-ml dose administered intradermally, or a subunit vaccine (15 μg HA/strain/0.5-ml dose) adjuvanted with MF59C.1 and administered intramuscularly. Blood samples were taken before and 21 ± 3 days post-vaccination. Anti-HA antibody titres were assessed using haemagglutination inhibition (HI) and single radial haemolysis (SRH) methods. We aimed to show that the intradermal vaccine was non-inferior to the adjuvanted vaccine.

**Results:**

A total of 795 participants were enrolled (intradermal vaccine n = 398; adjuvanted vaccine n = 397). Non-inferiority of the intradermal vaccine was demonstrated for the A/H1N1 and B strains, but not for the A/H3N2 strain (upper bound of the 95% CI = 1.53) using the HI method, and for all three strains by the SRH method. A *post-hoc *analysis of covariance to adjust for baseline antibody titres demonstrated the non-inferiority of the intradermal vaccine by HI and SRH methods for all three strains. Both vaccines were, in general, well tolerated; the incidence of injection-site reactions was higher for the intradermal (70.1%) than the adjuvanted vaccine (33.8%) but these reactions were mild and of short duration.

**Conclusions:**

The immunogenicity and safety of the intradermal seasonal influenza vaccine in the elderly was comparable with that of the adjuvanted vaccine. Intradermal vaccination to target the immune properties of the skin appears to be an appropriate strategy to address the challenge of declining immune responses in the elderly.

**Trial registration:**

ClinicalTrials.gov: NCT00554333.

## Background

The influenza A and B viruses are common respiratory pathogens, with estimated annual global attack rates of 5-10% in adults and 20-30% in children [1]. Seasonal influenza can be a severe disease. It has been estimated that in Europe between 40,000 and 220,000 excess deaths per year can be attributed to influenza, depending on the pathogenicity of the circulating virus [2]. Seasonal influenza affects all age groups, but the highest incidence of influenza-associated morbidity and mortality is seen in children aged 0-23 months and in adults over 65 years of age [3]. In the USA, influenza-associated deaths range between 30 and 150 per 100,000 individuals aged over 65 years [1], and it is estimated that ~90% of deaths due to seasonal influenza occur among people aged ≥ 65 years [1,4,5].

Annual vaccination continues to be the primary preventive measure against seasonal influenza. The World Health Organization (WHO) recommends vaccination of people at high risk of severe influenza or associated complications, including elderly people [1]. The seasonal influenza vaccine has been shown to be effective in reducing influenza-like illness confirmed by laboratory tests and influenza-associated morbidity and mortality in high-risk groups [6-8]. However, the immune response to the vaccine is lower in the elderly than in younger healthy adults [9], due to a decline in immune function with age ('immunosenescence') [10].

To compensate for the effects of immunosenescence, vaccines for elderly people must be adapted to optimise the immune responses to vaccination. Several approaches have been investigated, including use of an adjuvant [11,12], increasing the doses of antigens (e.g. 60 μg haemagglutinin [HA] of each component compared with the standard 15-μg dose) [13,14], and using new routes of administration. Several studies have looked at ways to improve immune responses to vaccination by delivering vaccine via the intradermal route rather than the intramuscular or subcutaneous routes [15,16]. The dermis has several unique properties that allow generation of powerful immune responses. In particular, it contains resident and blood-derived dendritic cells. These have an important role in the capture and presentation of antigens to the cells of the adaptive immune system; it also has a rich supply of blood and lymphatic vessels, allowing circulation of immune cells [15,16]. Studies with vaccines against a number of diseases such as hepatitis B and rabies have demonstrated that intradermal delivery can be an effective alternative route for vaccination [16].

Intanza^® ^15 μg (also known in some countries as IDflu^® ^15 μg) is the first intradermal influenza vaccine to be licensed for use in elderly people (>60 years of age); it received marketing authorisation in the European Union in 2009 for use in adults 60 years of age or older. Consistent with the WHO recommendations for seasonal influenza vaccines, it contains each of one A/H1N1 strain, one A/H3N2 strain, and one B strain [17]. Clinical studies in elderly people have shown that Intanza 15 μg is more immunogenic than the standard intramuscular vaccine [18,19].

The present study was the first to compare the immunogenicity of this intradermal vaccine with that of a licensed adjuvanted influenza vaccine that was also developed to increase immune responses in the elderly [20]. The primary objective of the study was to demonstrate that Intanza 15 μg is at least as immunogenic for the three virus strains as the adjuvanted influenza vaccine delivered via the intramuscular route in elderly volunteers.

## Methods

### Ethical approval of the study protocol

The study was approved by ethics committees in participating countries (Comité de Protection des Personnes, France; Ethics Committee of Universitair Ziekenhuis Antwerpen, Belgium), and also by the French Health Products Safety Agency (Agence Française de Sécurité Sanitaire des Produits de Santé, AFSSaPS) in France and by the Federal Agency for Medicines and Health Products (Federaal Agentschap voor Geneesmiddelen en Gezondheidsproducten, FAGG) in Belgium. The study was conducted in accordance with the Declaration of Helsinki and Good Clinical Practice. Participants gave written informed consent before enrolment. The study was registered on ClinicalTrials.gov; identifier: NCT00554333.

### Study design

This phase-III, multicentre, randomised, controlled, open-label, parallel-group study was carried out in adults aged 65 years or older who were recruited at three centres in Belgium and seven centres in France between October and December 2007. Participants attended the study centre on two occasions. At visit 1, participants who were eligible for inclusion were randomised to receive intradermal vaccine or an adjuvanted intramuscular influenza vaccine. Investigators were provided with a masked randomised list generated using a 1:1 ratio based on balanced permuted blocks stratified by centre. A pre-vaccination blood sample was taken and a single dose of allocated vaccine given. At visit 2, which was scheduled 21 ± 3 days post-vaccination, a second blood sample was taken for assessment of immunogenicity and collection of vaccine safety data.

### Participants

Individuals were invited to participate by the investigators via a phone call, an invitation letter, or during a consultation. Participants aged 65 years or older were eligible for enrolment in the study provided they met none of the exclusion criteria: acute febrile illness (oral temperature ≥ 37.5°C); systemic hypersensitivity to egg or chicken proteins or any of the vaccine constituents; thrombocytopenia or a bleeding disorder contraindicating intramuscular vaccination; and unstable chronic illness (defined as illness requiring hospitalisation or a clinically significant change in medication in the previous 12 weeks). Individuals were also excluded if they had: congenital or acquired immunodeficiency; received treatment with immunosuppressive therapy within the previous 6 months or long-term treatment with systemic corticosteroids; received blood or blood-derived products in the previous 3 months. Additional exclusion criteria were: current abuse of alcohol or drug addiction; any vaccination within the previous 4 weeks or planned vaccination within the 4 weeks after the first vaccination; seasonal influenza vaccination in the previous 6 months; or previous seasonal influenza vaccination by the intradermal route.

### Vaccines

Both vaccines were formulated according to the WHO recommendations for the 2007-2008 Northern Hemisphere influenza season: A/Solomon Islands/3/2006 (H1N1)-like strain; A/Wisconsin/67/2005 (H3N2)-like strain; and B/Malaysia/2506/2004-like strain.

The intradermal vaccine (Intanza^® ^15 μg; Sanofi Pasteur, Lyon, France) is an inactivated, split-virion influenza vaccine containing 15 μg HA/strain per 0.1-ml dose, all propagated in fertilised hen's eggs. The manufacturing process is based on that used for the intramuscular seasonal vaccine, with an additional concentration step to obtain a lower volume for intradermal use. The vaccine was administered in the deltoid region using a pre-filled intradermal micro-needle injection system (Becton Dickinson; Franklin Lakes, NJ, USA) [21].

The adjuvanted vaccine (Addigrip^®^/Fluad^®^, Novartis Vaccines and Diagnostics S.r.l., Origgio, Italy) is a subunit (HA and neuraminidase) influenza vaccine containing 15 μg HA/strain per 0.5-ml dose, all propagated in fertilised hen's eggs and adjuvanted with the squalene-based adjuvant MF59C.1. The vaccine was administered by injection into the deltoid muscle.

### Immunogenicity assessment

Consistent with previous studies of this intradermal vaccine, the primary immunogenicity endpoints were anti-HA antibody titres (geometric mean titre) for the three strains on day 21 post-vaccination [18,19]. Antibody titres were determined for each strain before and 21 days after vaccination by haemagglutination inhibition (HI) assay [22]. This was carried out in accordance with standard procedures at sanofi pasteur in which the antibody titre is the highest reciprocal dilution that induced complete inhibition of haemagglutination (1/dilution [1/dil]). The secondary endpoints were anti-HA antibody titres for the three strains assessed using the single radial haemolysis (SRH) method, in which anti-HA antibody titres are measured in square millimetres (mm^2^) [23]. SRH assays were performed by the National Institute for Biological Standards and Control (NIBSC, Hertfordshire, UK). HI and SRH assays are recognised by the European Medicines Agency (EMA) for the assessment of the immunogenicity of influenza vaccines [24,25].

The other secondary endpoints were anti-HA individual titre ratios (geometric mean titre ratio [GMTR], day 21/day 0) determined using the HI and SRH methods, the post-vaccination seroprotection rate (defined as the percentage of patients with anti-HA titre ≥ 40 [1/dil] or ≥ 25 mm^2 ^for the HI and SRH methods, respectively), and seroconversion or significant increase rate on day 21 according to HI and SRH methods. For the HI method, seroconversion was defined as post-vaccination anti-HA titre ≥ 40 (1/dil) for subjects with a pre-vaccination titre < 10 (1/dil) and a significant increase was defined as ≥ fourfold increase in anti-HA titre for subjects with a pre-vaccination titre ≥ 10 (1/dil). For the SRH method, seroconversion was defined as a post-vaccination anti-HA titre ≥ 25 mm^2 ^for subjects with a pre-vaccination titre ≤ 4 mm^2^, and a significant increase was defined as ≥ 1.5-fold increase from pre-to post-vaccination for subjects with a pre-vaccination titre > 4 mm^2 ^[24].

Compliance with EMA criteria (as defined for annual re-licensure trials) for the immunogenicity of influenza vaccines in elderly people for both the HI and SRH methods was also assessed: a GMTR >2, a seroprotection rate ≥ 60%, and a rate of seroconversion or significant titre increase ≥ 30% using either test [24].

### Safety assessments

Participants used diary cards to record details of solicited injection-site reactions (pain, erythema, swelling, induration, ecchymosis, pruritus) and solicited systemic reactions (fever [rectal equivalent temperature ≥ 38.0°C], headache, malaise, myalgia, shivering) occurring within 7 days of vaccination. Participants also recorded unsolicited adverse events and serious adverse events occurring within 21 days of vaccination. If the investigators felt that adverse events were possibly, probably or definitely related to the study vaccine, they were considered to be 'adverse reactions'. Occurrence of solicited adverse reactions according to EMA criteria was also assessed in the three days after vaccination: injection-site induration >5 cm observed for > 3 consecutive days; injection-site ecchymosis; pyrexia (rectal equivalent temperature > 38.0°C) for ≥ 24 hours; malaise; and shivering [24].

### Statistical methods

Three sets of participants were defined for the assessment of immunogenicity, and one set for the safety analysis (Table 1).

**Table 1:** Description of analysis sets and numbers (percentage) of subjects by vaccine group; subjects were analysed according to the vaccine they received except for the randomised set

	Intradermal vaccine, n (%)	Adjuvanted vaccine, n (%)
Randomised set^a^	398 (100)	397 (100)
Full analysis set^b^	395 (99.2)	395 (99.5)
Per protocol set^c^		
HI method^d^	390 (98.0)	385 (97.0)
SRH method^e^	389 (97.7)	382 (96.2)
Other immunogenicity analysis set for EMA immunogenicity criteria ^f^		
HI method (full analysis set)		
A/H1N1	395 (99.2)	395 (99.5)
A/H3N2	395 (99.2)	395 (99.5)
B	395 (99.2)	395 (99.5)
SRH method^g^		
A/H1N1	391 (98.2)	389 (98.0)
A/H3N2	391 (98.2)	388 (97.7)
B	391 (98.2)	389 (98.0)
Safety set^h^	398 (100)	397 (100)

^a ^All individuals randomised who received a study vaccine.

^b ^All randomised individuals who received a study vaccine and who had post-vaccination immunogenicity results. Intradermal vaccine group: one participant withdrew due to a serious adverse event, and insufficient blood samples for testing were noted for two participants; adjuvanted vaccine group: one participant withdrew consent and an insufficient blood sample for testing was noted for one participant.

^c ^All randomised individuals except those with protocol violations that could interfere with the immunogenicity evaluation.

^d ^Additional participants with at least one protocol deviation excluded: intradermal vaccine group n = 5, adjuvanted vaccine group n = 10.

^e ^Additional excluded participants with insufficient blood samples: intradermal vaccine group n = 1, adjuvanted vaccine group n = 3.

^f ^Participants who received a study vaccine and for whom pre-and post-vaccination titres on days 0 and 21 were available for each strain.

^g ^Additional participants excluded because of missing pre-and/or post-vaccination SRH method results: intrademal vaccine group n = 4 for all three strains; adjuvanted vaccine group n = 6 for A/H1N1 and B and n = 7 for A/H3N2.

^h ^Participants who received a study vaccine and who had post-vaccination safety data.

The primary hypothesis was that the immunogenicity of the intradermal vaccine would be non-inferior to that of the adjuvanted vaccine for each virus strain in terms of antibody titres using the HI method. In the study, non-inferiority was tested with HI and SRH methods, and was defined as the upper bound of the 95% confidence intervals (CIs) around the post-vaccination ratios of geometric mean titres (GMT) (adjuvanted vaccine/intradermal vaccine) being < 1.5 for all three strains in the per protocol set [26].

If non-inferiority was achieved for the three strains, the superiority of the intradermal vaccine in the full analysis set was also investigated. Superiority was defined as the upper bound of the 95% CI being < 1 for at least two of the three strains. The primary analysis model did not include adjustments. Baseline antibody titres between the two vaccine groups were different so, to improve the accuracy of the estimates, a *post-hoc *analysis of covariance (ANCOVA model with group and pre-vaccination titre; no interaction) was performed to compare post-vaccination results adjusted according to pre-vaccination levels [26].

To demonstrate non-inferiority of immune responses induced by the intradermal vaccine compared with the adjuvanted vaccine with a power of >90% and to allow for 10% of participants not being evaluable, enrolment of 395 participants was planned for each group to provide 355 evaluable participants per group in the per protocol analysis. The analysis of the secondary endpoints included calculation of differences or ratios (and 95% CI) between groups, and safety endpoints were analysed using descriptive statistics.

## Results

### Participants

A total of 795 participants were enrolled in the study (intradermal vaccine, n = 398; adjuvanted vaccine, n = 397; Table 1). Two participants withdrew from the study. One participant in the intradermal vaccine group had a cardiac arrest that led to death on day 20 (this event was considered to be unrelated to the vaccine; this 82-year old male had a history of coronary insufficiency and chronic obstructive pulmonary obstruction and several risk factors for myocardial infarction, including hypertension and past smoking). One participant in the adjuvanted vaccine group withdrew consent. After the second study visit, blood samples for analysis of immunogenicity were available for 395 participants in each group. All sera were tested by the HI method and most of them (with adequate samples) were evaluated by the SRH method (Table 1).

The demographic characteristics of the participants randomised in the two groups were comparable (Table 2). The study involved 370 men (46.5%) and 425 women (53.5%), with a mean age of 74.3 ± 6.4 years (range, 60.3-94.2 years). One individual aged 60.3 years and who was therefore below the age for inclusion was randomised by error and received the intradermal vaccine. This individual was excluded from the per protocol analyses but included in all other analyses. Most participants in both groups (89.9%) had past and/or current significant medical conditions, but only 52.7% of participants had underlying diseases identified as risk factors for influenza-related morbidity (mainly heart diseases). Overall, 72.5% of participants had been vaccinated against influenza in the previous season (2006-2007).

**Table 2:** Demographic data and baseline characteristics (randomised set)

	Total	Intradermal vaccine	Adjuvanted vaccine
	N = 795	N = 398	N = 397
Mean age (years; SD)	74.3 (6.4)	73.9 (6.3)	74.7 (6.6)
- over 75 (n; %)	329 (41.4)	154 (38.7)	175 (44.1)
Males (n; %)	370 (46.5)	194 (48.7)	176 (44.3)
Mean body mass index (kg/m^2^; SD)	27.1 (4.5)	27.0 (4.5)	27.2 (4.5)
Past or current significant medical history (n; %)	715 (89.9)	358 (89.9)	357 (89.9)
- risk factor ^a ^(n; %)	419 (52.7)	216 (54.3)	203 (51.1)
- history of allergy ^b ^(n; %)	67 (8.4)	29 (7.3)	38 (9.6)
Last influenza vaccination ^c^			
- previous season 2006-2007 (n; %)	576 (72.5)	287 (72.1)	289 (72.8)
- prior to previous season (n; %)	57 (7.2)	26 (6.5)	31 (7.8)

Percentages are based on the number of randomised subjects having available data

^a ^At least one of the following: lung disease, heart disease, diabetes, renal disease, neurological abnormality or other significant medical history such as HIV, cancer, hepatitis (A, B, C), epilepsy, auto-immune diseases or blood disorders reported during the medical history review

^b ^At least one allergic condition reported as medical history

^c ^Percentages are calculated based on the number of randomised subjects who had previously been vaccinated against influenza and who had available data. 7 subjects (4 from the intradermal vaccine group and 3 from the adjuvanted vaccine group) had previously been vaccinated against influenza by the intradermal route

### Immunogenicity

The geometric mean antibody titres induced by the intradermal and adjuvanted vaccines for all three virus strains by both the HI and SRH methods are summarised in Table 3. For the primary objective of non-inferiority of the intradermal vaccine immunogenicity compared with that for the adjuvanted vaccine assessed by the HI method in the per protocol population, non-inferiority was not demonstrated for the A/H3N2 strain (upper bound of the 95% CI = 1.53) (Table 3). Non-inferiority was demonstrated for all three strains by the SRH method (Table 3). Similar results for the three strains were observed in the full analysis set with both the HI and the SRH methods (ratios [adjuvanted vaccine/intradermal vaccine] of post-vaccination GMT: A/H1N1 1.12, A/H2N3 1.31, B 1.07; and A/H1N1 1.16, A/H2N3 1.18, B 1.05, respectively). Non-inferiority of the intradermal vaccine was not demonstrated for all three strains by the HI method, so the superiority of the vaccine was not tested. Superiority of the intradermal vaccine by the SRH method was tested on the full analysis set, but superior immunogenicity was not demonstrated for any of the virus strains.

**Table 3:** Post-vaccination geometric mean titres (GMT) and ratios of GMT (adjuvanted vaccine/intradermal vaccine) for the three virus strains on day 21 in the per protocol set assessed by the haemagglutinin inhibition (HI) and single radial haemolysis (SRH) methods

		HI method (1/dil)			**SRH method (mm**^**2**^)		
		
		Intradermal vaccine (N = 390)	Adjuvanted vaccine (N = 385)		Intradermal vaccine (N = 389)	Adjuvanted vaccine (N = 382)	
Virus strain	Pre or Post vaccination	GMT [95% CI]		Ratios of GMT [95% CI]	GMT [95% CI]		**Ratios **of **GMT** [95% CI]
A/H1N1	Pre-	13.3 [12.1;14.6]	13.5 [12.3;14.9]	--	7.7 [6.9;8.6]	8.0 [7.2;8.9]	--
	Post-	108.3 [95.4;123.0]	122.1 [109.1;136.7]	1.13 [0.95;1.34]*	46.4 [41.6;51.8]	53.9 [49.0;59.3]	1.16 [1.00;1.34]*
A/H3N2	Pre-	60.3 [52.2;69.7]	69.4 [59.5;81.0]	--	11.0 [9.8;12.5]	12.7 [11.2;14.3]	--
	Post-	259.9 [233.5;289.3]	341.4 [306.7;380.1]	1.31 [1.13;1.53]	39.3 [35.6;43.3]	46.2 [42.1;50.7]	1.18 [1.03;1.34]*
B	Pre-	15.0 [13.7;16.4]	16.5 [15.1;18.1]	--	29.2 [25.6;33.2]	28.0 [24.5;32.1)	--
	Post-	36.9 [33.6;40.5]	39.9 [36.4;43.8]	1.08 [0.95;1.23]*	66.5 [60.8;72.8]	68.9 [62.9;75.3]	1.03 [0.91;1.17]*

*Non-inferiority of the intradermal vaccine was demonstrated if the upper bound of the 95% CI for the ratios of GMT was < 1.5.

For the A/H1N1 strain (HI method), baseline and post-vaccination antibody titres were correlated (Pearson's correlation coefficient ρ = 0.5 in per protocol set) and baseline antibody titres were slightly different in the two groups (pre-vaccination GMTR of 1.15 in per protocol set). Therefore a *post-hoc *ANCOVA adjusting for baseline values was carried out. Non-inferiority of the intradermal vaccine compared with the adjuvanted vaccine for all three virus strains by HI and SRH methods was observed (Table 4).

**Table 4:** *Post-hoc *analysis in which post-vaccination geometric mean titres (GMTs) were adjusted according to pre-vaccination titres and ratios of GMT (adjuvanted vaccine/intradermal vaccine) for the three virus strains on day 21 in the per protocol set assessed by haemagglutinin inhibition (HI) and single radial haemolysis (SRH) methods

	HI method (1/dil)			**SRH method (mm**^**2**^)		
	Intradermal vaccine (N = 390)	Adjuvanted vaccine (N = 385)		Intradermal vaccine (N = 389)	Adjuvanted vaccine (N = 382)	
Virus strain	Post-vaccination GMT [95% CI]		Ratios of GMT [95% CI]	Post-vaccination GMT [95% CI]		Ratios of GMT [95% CI]
A/H1N1	108.8	121.6	1.12	47.1	54.0	1.15
	[97.4;121.5]	[108.8;135.9]	[0.95;1.31]*	[42.8;51.9]	[49.0;59.6]	[1.00;1.32]*
A/H3N2	266.5	332.8	1.25	40.3	45.3	1.12
	[243.1;292.3]	[303.3;365.2]	[1.10;1.42]*	[37.0;44.0]	[41.5;49.4]	[0.99;1.27]*
B	37.9	38.9	1.03	65.8	69.8	1.06
	[35.0;41.0]	[35.9;42.1]	[0.92;1.15]*	[61.0;71.1]	[64.6;75.4]	[0.95;1.18]*

* Non-inferiority of the ID vaccine was demonstrated if the upper bound of the 95% CI for the ratios of GMT was < 1.5

**Figure 1:**
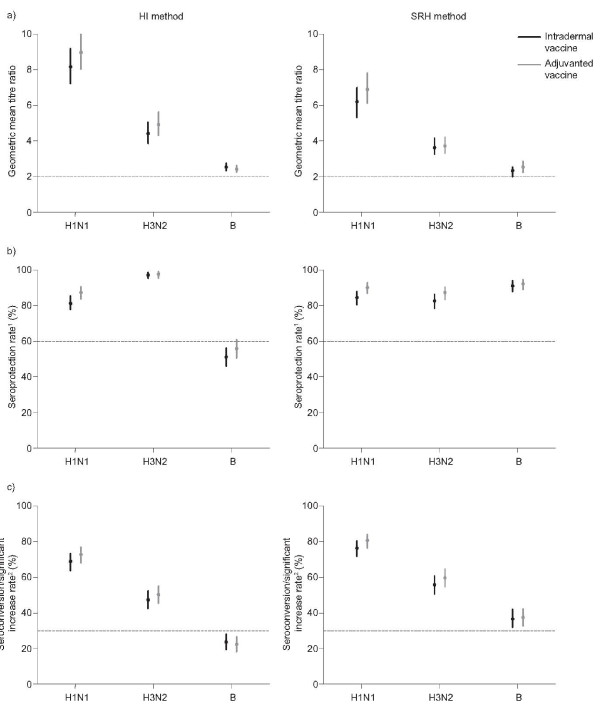
**Comparison of European Medicines Agency (EMA) immunogenicity variables for the intradermal and adjuvanted influenza vaccines assessed by haemagglutinin inhibition (HI; left-hand column) and single radial haemolysis (SRH; right-hand column) methods**. Analyses were carried out with the other immunogenicity analysis set (participants who received a study vaccine and for whom pre-and post-vaccination titres on days 0 and 21 were available for each strain). The horizontal line indicates the EMA threshold for each variable. (a) Ratio of individual post-and pre-vaccination geometric mean titres (GMTR); (b) Post-vaccination seroprotection rate^1 ^(%); (c) Seroconversion/significant increase rate^2 ^(%). *: statistically significantly difference between the groups (adjuvanted vaccine -intradermal vaccine) was observed for the A/Solomon (HINI) strain using the full analysis set: 5.8% (95% CI: 1.1-10.5). ^1^Seroprotection: percentage of participants with anti-HA titre ≥ 40 [1/dil] or ≥ 25 mm^2 ^for HI and SRH methods, respectively. ^2^Seroconversion: anti-HA post-vaccination titre ≥ 40 (1/dil; HI method) or ≥ 25 mm^2 ^(SRH method) for participants with a pre-vaccination anti-HA individual titre < 10 (1/dil; HI method) or ≥ 4 mm^2 ^(SRH method). Significant increase: ≥ fourfold increase (HI method) or ≥ 1.5-fold increase (SRH method) from pre-to post-vaccination anti-HA individual titre for participants with a pre-vaccination anti-HA individual titre ≥ 10 (1/dil - HI method) or > 4 mm^2 ^(SRH method).

In the immunogenicity assessment by the HI method, the results for the A/H1N1 and A/H3N2 strains with both vaccines satisfied all three EMA criteria, and the results for the B strain with both vaccines satisfied the EMA criterion for GMTR (Figure 1). When immunogenicity was assessed by the SRH method, the results for all three strains with both vaccines satisfied all EMA immunogenicity criteria (Figure 1). There were no significant differences between the two vaccine groups in GMTRs, seroprotection rates, and seroconversion/significant increase rates for the three virus strains by either assay, with the exception of the seroprotection rate for the A/H1N1 strain (Figure 1). Although the seroprotection rates for the A/H1N1 strain were high in both the intradermal and adjuvanted groups (HI method: 81.3% and 87.1%, respectively; SRH method: 84.3% and 90.1%, respectively), they were higher in the adjuvanted vaccine group (difference between groups: HI method: 5.8% [95% CI: 0.7-10.9]; SRH method: 5.8% [95% CI: 1.1-10.5]).

### Safety

**Figure 2:**
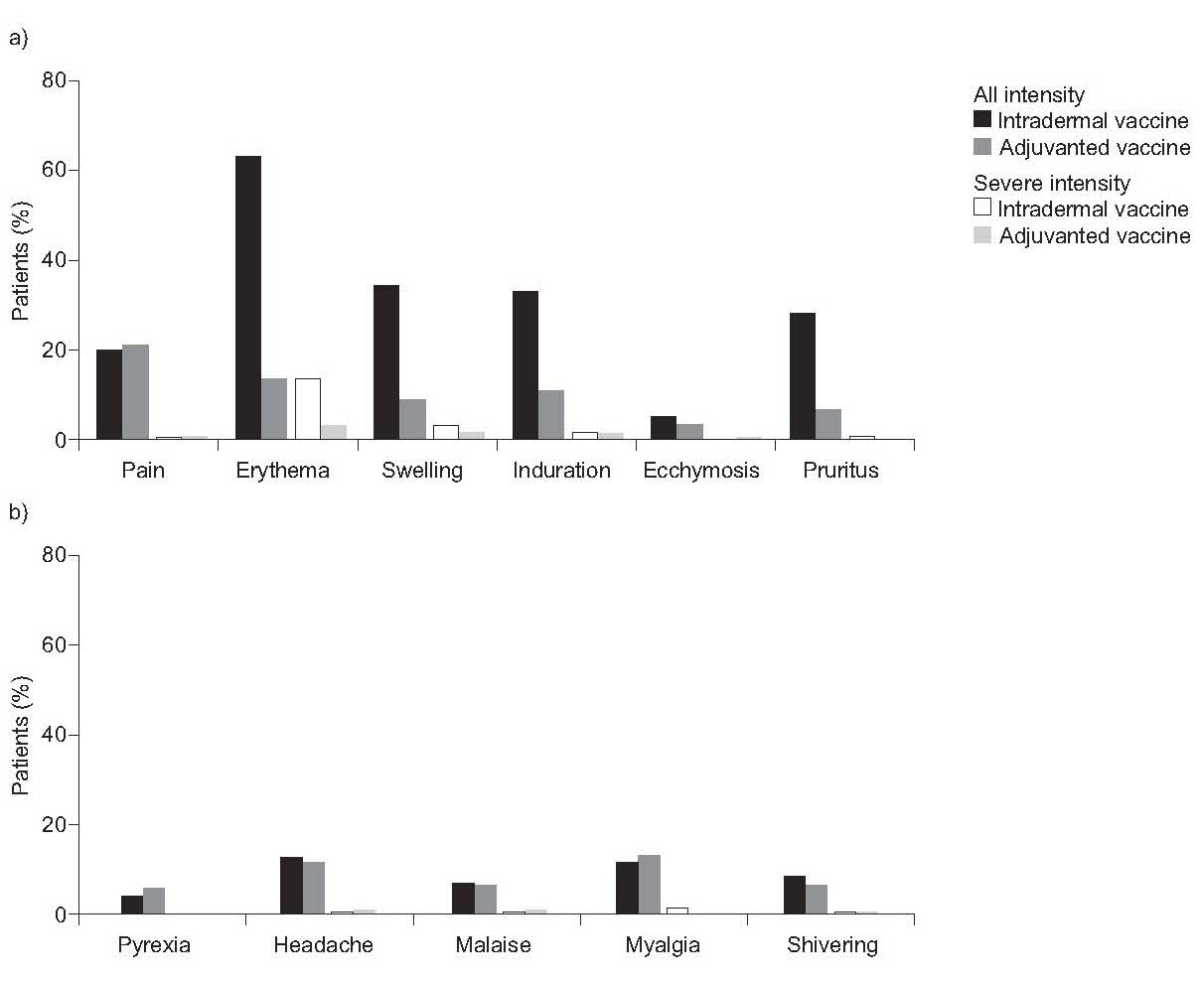
**Summary of safety assessments with the intradermal and adjuvanted vaccines on days 0-7 post-vaccination**. (a) Incidence of all and of severe solicited injection-site reactions. (b) Incidence of solicited systemic reactions

The incidence of reactions within three days after the first vaccination listed in the EMA guideline (most commonly shivering and malaise) was similar in the intradermal and adjuvanted vaccine groups (Table 5). Induration of >5 cm that lasted for >3 days was not observed in either group. More participants in the intradermal vaccine group reported at least one solicited injection-site reaction (70.1%) compared with the adjuvanted vaccine group (33.8%). Erythema was reported more frequently in the intradermal vaccine group (63.1% vs. 13.4%). Swelling (34.2% vs. 8.6%), induration (32.9% vs. 10.6%) and pruritus (28.1% vs 6.5%) were also more frequently reported but at a lower incidence than for erythema. The proportion of participants who reported pain (19.8% vs. 20.9%) or ecchymosis (4.8% vs. 3.0%) was comparable between the two vaccine groups (Figure 2a). Most of the solicited injection-site reactions were of mild intensity or < 2.5 cm, and most occurred between day 0 and day 3 post-vaccination and lasted ≤ 3 days without treatment. Injection-site erythema lasted ≥ 8 days in 22 participants in the intradermal vaccine group compared with 4 participants in the adjuvanted vaccine group.

**Table 5:** Incidence of solicited adverse reactions as defined by the EMA in the first three days after vaccination

	Intradermal vaccine	Adjuvanted vaccine
	(N = 398)	(N = 397)
	n	n
	(%; [95% CI])	(%; [95% CI])
≥ 1 adverse reaction	51	55
	(12.8 [9.7;16.5])	(13.9 [10.6;17.6])
Injection-site indurationa	0	0
	(0 [0.0;0.9])	(0 [0.0;0.9])
Injection-site ecchymosis	13	12
	(3.3 [1.8;5.5])	(3.0 [1.6;5.2])
Pyrexia^b^	6	12
	(1.5 [0.6;3.3])	(3.0 [1.6;5.2])
Malaise	19	20
	(4.8 [2.9;7.4])	(5.0 [3.1;7.7])
Shivering	24	23
	(6.0 [3.9;8.8])	(5.8 [3.7;8.6])

^a ^> 5 cm for > 3 consecutive days.

^b ^Rectal equivalent temperature > 38.0°C for ≥ 24 hours.

Overall, 99 participants (24.9%) in the intradermal vaccine group and 96 participants (24.2%) in the adjuvanted vaccine group reported at least one solicited systemic reaction in the first 7 days after vaccination. The incidence of each solicited systemic reaction was comparable for the two vaccine groups (Figure 2b). Most reactions were of mild intensity. Only 4.0% of the participants in the intradermal vaccine group and 5.8% of those in the adjuvanted vaccine group had a fever. In both groups pyrexia was, in general, ≤ 38.5°C (3.3% and 4.0% of subjects, respectively) and none were > 39.6°C, During the 21 days post-vaccination, there were no differences between the intradermal and adjuvanted vaccine groups in the proportion of participants reporting systemic adverse events (31.9% *vs *32.5%; severe 3.3% *vs *2.5%) or systemic adverse reactions (26.4% *vs *25.9%; severe 2.0% *vs *1.8%). No unsolicited adverse events occurred within 30 minutes of vaccination in either group. Six participants reported serious adverse events, two of which were considered by the investigator to be possibly related to vaccination: a participant in the intradermal vaccine group presented with pneumonia one day after vaccination, and a participant in the adjuvanted vaccine group presented with facial herpes zoster three days after vaccination.

## Discussion

This study showed that, in elderly volunteers, the immunogenicity of the intradermal seasonal influenza vaccine is largely comparable with that of the adjuvanted influenza vaccine. The two vaccines induced similar levels of anti-HA antibodies as assessed by the SRH method. Non-inferiority of the intradermal vaccine compared with the adjuvanted vaccine was demonstrated for the A/H1N1 and B strains with the HI method and for all three strains with the SRH method. Both vaccines satisfied the EMA immunogenicity criteria for influenza vaccines in the elderly. Both vaccines were developed to address the challenge of immunosenescence. The aim of the adjuvant in the seasonal influenza vaccine is to increase the immunogenicity in elderly people [20], whereas this intradermal vaccine reaches the same goal by reliably delivering the vaccine into the rich immune environment of the dermis a using a micro-needle injection system [15,16,21].

The HI assay is the most widely used for surveillance of the influenza virus and for assessing responses to vaccines in clinical trials [25]. The HI and SRH methods provide serological correlates of protection [25], and are recognised by the EMA for the evaluation of influenza vaccines [24]. The good agreement between the results from the HI and SRH tests for the A strains reported previously [27] was also observed in the present study because the results from both tests were comparable for the A strains for both vaccines. Results from the SRH assay satisfied all three EMA immunogenicity criteria for both vaccines, whereas the HI assay results satisfied only the GMTR criterion, confirming the higher sensitivity of the SRH assay for influenza B strains [25,27].

Due to the epidemiology of influenza and vaccination programmes in industrialised countries, few elderly people are seronegative to circulating seasonal influenza antigens [26]. Beyer et al. demonstrated that pre-vaccination antibody titres can have an important influence on the results of immunogenicity studies with influenza vaccines (e.g. underestimation of antibody responses) [28]. They proposed that post-vaccination results should be adjusted according to individual pre-vaccination titres so as to provide a more informative measure of serological responses to influenza vaccines [28]. In the present study, baseline GMTs for all strains (but particularly the A/H3N2 strain) were slightly lower in the intradermal vaccine group than in the adjuvanted vaccine group. Although the A/H3N2 strain failed to meet the criterion for non-inferiority when assessed by the HI method in the primary analysis, the *post*-*hoc *covariance analysis to adjust for pre-vaccination anti-HA titres demonstrated that the intradermal vaccine was non-inferior to the adjuvanted vaccine for all three virus strains assessed by the HI method.

In general, the intradermal and adjuvanted vaccines were well tolerated, with comparable proportions of participants reporting solicited systemic reactions in the two groups. The two vaccines were also comparable according to the EMA criteria for the safety of influenza vaccines. The micro-needle injection system used to deliver Intanza 15 μg has advantages over the adjuvanted vaccine from the perspective of local safety. The needle penetrates only 1.5 mm into the skin, so the risk of damage to nerves or veins inherent with intramuscular injection is eliminated [19,21]. The increased rate of injection-site erythema compared with intramuscular vaccination has been reported in studies with this intradermal vaccine in elderly volunteers [18,19]. The injection of vaccine just below the skin surface means that local reactions are more readily and more frequently visible than after injection of vaccine deeper into the muscle even if the symptoms ere mainly mild and of short duration[16,29,30]. Importantly, injection-site reactions in the intradermal group were not associated with a higher incidence or severity of injection-site pain, and most reactions lasted for ≤ 3 days.

## Conclusion

The present study demonstrated that the immunogenicity and safety of a new intradermal seasonal influenza vaccine for use in the elderly, Intanza 15 μg, is largely comparable with that of the adjuvanted vaccine that has been licensed for use in elderly people in Europe for several years. Thus, intradermal vaccination to target the immune properties of the skin appears to be an appropriate strategy to address the challenge of declining immune responses in the elderly.

## Competing interests

PVD declared that he acts as chief and principal investigator for clinical trials conducted on behalf of the University of Antwerp, for which the University obtains research grants from vaccine manufacturers; speaker's fees for presentations on vaccines are paid directly to an educational fund held by the University of Antwerp.

RA declared no competing interests

FK declared no competing interests

AF, PR, SIS and ST declared that they work for Sanofi Pasteur MSD who commercialise the seasonal intradermal vaccine

GM and EL declared that they worked for Sanofi Pasteur MSD who commercialise the seasonal intradermal vaccine at the time the clinical trial was performed and the results analysed.

## Authors' contributions

All authors have critically reviewed the drafts and have read and approved the final manuscript. In addition, PVD participated in the data collection, data analysis, and interpretation of the data; RA participated in data collection; FK participated in the data collection; AF participated in the trial design, data analysis, and interpretation of the data and was the trial medical officer; PR, ST and SIS participated in the trial design, data analysis, and interpretation of the data; GM and EL participated in the trial design, the selection of study sites, logistic details of the conduct of the study, data analysis, and interpretation of the data.

## Pre-publication history

The pre-publication history for this paper can be accessed here:

http://www.biomedcentral.com/1471-2334/10/134/prepub

## References

[B1] World Health Organization Influenza Vaccines. WHO position paper Wkly Epidemiol Rec 2005 80 277 28716171030

[B2] ECDC, Seasonal Human Influenza and Vaccination - The Facts. European Centre for Disease Prevention and Control http://ecdc.europa.eu/en/healthtopics/Documents/0712_seasonal_human_influenza_vaccination.pdf

[B3] Harper SA Fukuda K Uyeki TM Cox NJ Bridges CB Prevention and control of influenza. Recommendations of the Advisory Committee on Immunization Practices (ACIP) MMWR Recomm Rep 2005 54 RR-8 1 40 16086456

[B4] Poland G Morse D Improving the public health: The U.S. recommendation for universal influenza immunization Vaccine 2010 28 2799 2800 10.1016/j.vaccine.2010.03.002 20346856

[B5] Thompson WW Shay DK Weintraub E Mortality associated with influenza and respiratory syncytial virus in the United States JAMA 2003 289 179 186 10.1001/jama.289.2.179 12517228

[B6] Govaert TM Thijs CT Masurel N Sprenger MJ Dinant GJ Knottnerus JA The efficacy of influenza vaccination in elderly individuals. A randomised double-blind placebo-controlled trial JAMA 1994 272 1661 1665 10.1001/jama.272.21.1661 7966893

[B7] Gross PA Quinann GV Jr Weksler ME Gaerlan PF Denning CR Immunization of elderly people with high doses of influenza vaccine J Am Geriatr Soc 1988 36 209 212 3339228 10.1111/j.1532-5415.1988.tb01802.x

[B8] Nichol KL The efficacy, effectiveness and cost-effectiveness of inactivated influenza virus vaccines Vaccine 2003 21 1769 1775 10.1016/S0264-410X(03)00070-7 12686092

[B9] Goodwin K Viboud C Simonsen L Antibody response to influenza vaccination in the elderly: a quantitative review Vaccine 2006 24 1159 1169 10.1016/j.vaccine.2005.08.105 16213065

[B10] Weinberger B Herndler-Brandstetter D Schwanninger A Weiskopf D Grubeck-Loebenstein B Biology of immune responses to vaccines in elderly persons Clin Infect Dis 2008 46 1078 1084 10.1086/529197 18444828

[B11] Guy B The perfect mix: recent progress in adjuvant research Nat Rev Microbiol 2007 5 505 517 10.1038/nrmicro1681 17558426

[B12] O'Hagan DT MF59 is a safe and potent vaccine adjuvant that enhances protection against influenza virus infection Expert Rev Vaccines 2007 6 699 710 10.1586/14760584.6.5.699 17931151

[B13] Palache AM Beyer WE Sprenger MJ Antibody response after influenza immunization with various vaccine doses: a double-blind, placebo-controlled, multi-centre, dose-response study in elderly nursing-home residents and young volunteers Vaccine 1993 11 3 9 10.1016/0264-410X(93)90333-S 8427034

[B14] Falsey AR Treanor JJ Tornieporth N Capellan J Gorse GJ Randomized, double-blind controlled phase 3 trial comparing the immunogenicity of high-dose and standard-dose influenza vaccine in adults 65 years of age and older J Infect Dis 2009 200 172 180 10.1086/599790 19508159

[B15] Lambert PH Laurent PE Intradermal vaccine delivery: will new delivery systems transform vaccine administration? Vaccine 2008 26 3197 3208 10.1016/j.vaccine.2008.03.095 18486285

[B16] Nicolas JF Guy B Intradermal, epidermal and transcutaneous vaccination: from immunology to clinical practice Expert Rev Vaccines 2008 7 1201 1214 10.1586/14760584.7.8.1201 18844594

[B17] Intanza^® ^Summary of Product Characteristics 2009 Sanofi Pasteur MSD, Lyon, France

[B18] Holland D Booy R De Looze F Intradermal influenza vaccine administered using a new microinjection system produces superior immunogenicity in elderly adults: a randomized controlled trial J Infect Dis 2008 198 650 658 10.1086/590434 18652550

[B19] Arnou R Icardi G De Decker M Intradermal influenza vaccine for older adults: a randomized controlled multicenter phase III study Vaccine 2009 27 7304 7312 10.1016/j.vaccine.2009.10.033 19849996

[B20] Podda A The adjuvanted influenza vaccines with novel adjuvants: experience with the MF59-adjuvanted vaccine Vaccine 2001 19 2673 2680 10.1016/S0264-410X(00)00499-0 11257408

[B21] Laurent PE Bonnet S Alchas P Evaluation of the clinical performance of a new intradermal vaccine administration technique and associated delivery system Vaccine 2007 25 8833 8842 10.1016/j.vaccine.2007.10.020 18023942

[B22] Palmer DF Dowdle WR Coleman MT Shild GC Advanced laboratory techniques for influenza diagnosis: procedural guide. Hemagglutination inhibition test 1975 Atlanta: US Department of Health, Education, and Welfare, Public Health Service 25 62

[B23] Schild GC Pereira MS Chakraverty P Single-radial-haemolysis: a new method for the assay of antibody to influenza haemagglutinin. Applications for diagnosis and seroepidemiologic surveillance of influenza Bull World Health Organ 1975 52 43 50 1082381 PMC2366333

[B24] Committee for Proprietary Medicinal Products Note for Guidance on Harmonisation of Requirements for Influenza Vaccines 1997 CPMP/BWP/214/96.

[B25] Wood JM Newman RW Ploss K The use of correlates of immunity in European Union licensing of influenza vaccines Dev Biol (Basel) 2003 115 9 16 15088770

[B26] Center for Biologics Evaluation and Research, US Food and Drug Administration Clinical data needed to support the licensure of seasonal inactivated influenza vaccines http://www.fda.gov/downloads/BiologicsBloodVaccines/GuidanceComplianceRegulatoryInformation/Guidances/Vaccines/ucm091990.pdf Accessed 19 April 2010.

[B27] Wood JM Gaines-Das RE Taylor J Chakraverty P Comparison of influenza serological techniques by international collaborative study Vaccine 1994 12 167 174 10.1016/0264-410X(94)90056-6 8147099

[B28] Beyer WE Palache AM Lüchters G Nauta J Osterhaus AD Seroprotection rate, mean fold increase, seroconversion rate: which parameter adequately expresses seroresponse to influenza vaccination? Virus Res 2004 103 125 132 10.1016/j.virusres.2004.02.024 15163500

[B29] Belshe RB Newman FK Cannon J Serum antibody responses after intradermal vaccination against influenza N Engl J Med 2004 351 2286 2294 10.1056/NEJMoa043555 15525713

[B30] Kenney RT Frech SA Muenz LR Villar CP Glenn GM Dose sparing with intradermal injection of influenza vaccine N Engl J Med 2004 351 2295 2301 10.1056/NEJMoa043540 15525714

